# A UV-DOAS-Based Multi-Scale Interaction Attention Network for Simultaneous Retrieval of NO and NO_2_ in Gas Mixtures

**DOI:** 10.3390/s26113461

**Published:** 2026-05-30

**Authors:** Yuwei Mu, Hua Wen

**Affiliations:** Purification Equipment Research Institute of CSSC, Handan 056000, China; wenhua_prof@163.com

**Keywords:** nitric oxide, nitrogen dioxide, gas mixtures, concentration retrieval, attention mechanism, deep learning, chemometric spectroscopy

## Abstract

Accurate monitoring of nitrogen oxides (NOx) is essential due to their adverse effects on environmental quality and public health. To address the spectral overlap between NO and NO_2_ in ultraviolet differential optical absorption spectroscopy (UV-DOAS), we propose a multi-scale dual-branch interaction attention network (MDIAN) for simultaneous concentration retrieval in gas mixtures. The model employs a dual-branch multi-scale convolutional architecture to extract local narrow-band absorption details and broad spectral profile features. A cross-attention mechanism is introduced to enable feature interaction between the NO and NO_2_ branches. A bidirectional long short-term memory (Bi-LSTM) network is further incorporated to model contextual dependencies along the wavelength dimension, enabling joint regression of both target gases. Experimental results show that the proposed model achieves mean absolute errors (MAE) of 0.076 ppm for NO and 0.062 ppm for NO_2_, with coefficients of determination (R^2^) of 0.9998 for both gases, outperforming traditional regression methods and baseline deep learning models. The uncertainties are 0.69% and 0.76%, respectively, and the inference time per sample ranges from 48.9 to 74.5 ms. These results indicate that MDIAN achieves a favorable balance among accuracy, stability, and real-time performance, offering a promising approach for intelligent monitoring of complex gas mixtures using UV-DOAS.

## 1. Introduction

Nitrogen oxides (NOx) are major atmospheric pollutants mainly generated by the high-temperature combustion of fossil fuels, such as motor vehicle emissions, thermal power generation, industrial boilers, and chemical manufacturing [1,2]. Nitric oxide (NO) is a key precursor to photochemical smog and can contribute to ozone pollution while impairing respiratory health [3]. Nitrogen dioxide (NO_2_) is a strongly irritating gas, and long-term exposure to it is associated with increased risks of respiratory and cardiovascular diseases [4,5]. Accordingly, the rapid and accurate quantification of NO and NO_2_ is of great practical importance for environmental quality assessment, emission monitoring, and public health protection [6].

To address this measurement need, ultraviolet differential optical absorption spectroscopy (UV-DOAS) has become a mainstream technique due to its advantages of non-contact measurement, high sensitivity, and real-time in situ monitoring [7,8]. This technique is based on the Beer–Lambert law and retrieves gas concentrations by analyzing the characteristic absorption of molecules in the ultraviolet spectral region [9,10]. However, NO and NO_2_ exhibit severe spectral overlap in the 200–230 nm ultraviolet range, which introduces significant cross-interference. Therefore, NO and NO_2_ were selected not only for their environmental relevance. They also provide a representative overlapping gas pair for investigating simultaneous UV-DOAS concentration retrieval under cross-interference. Such overlap makes it difficult for conventional DOAS methods to separate the contributions of the two gases, thereby reducing measurement accuracy. Traditional approaches, such as least squares fitting and multiple linear regression, have been employed to address this issue [11]. However, these linear models have limited ability to handle complex nonlinear interference.

In recent years, the rapid development of deep learning has provided new avenues for spectral analysis [12,13,14]. Convolutional neural networks (CNNs) can automatically extract local spectral features [15,16], while recurrent neural networks (RNNs) and their variants, including long short-term memory (LSTM) and bidirectional LSTM (Bi-LSTM), are well suited for modeling spectral data with sequential characteristics [17,18]. In addition, attention mechanisms enable models to dynamically focus on informative spectral regions, thereby enhancing representation capability [19,20]. In the field of gas detection, increasing efforts have been devoted to integrating deep learning with spectroscopic techniques. For example, Gao et al. developed a UV-DOAS system incorporating a convolutional attention neural network (CANN) for trace-level detection of multiple atmospheric pollutants [21]. Peng et al. proposed a two-stage framework combining Bi-LSTM and attention mechanisms to effectively address spectral separation and concentration estimation in NO–SO_2_ gas mixtures [22]. Xie et al. further achieved ppb-level detection of NH_3_, SO_2_, and NO through multi-dimensional spectral fusion [23]. Despite these advances, existing deep learning approaches still have limitations in multi-scale feature representation, overlap-interference suppression, and the balance between accuracy and real-time performance.

To address the aforementioned challenges, this study proposes a multi-scale dual-branch interaction attention network (MDIAN) for the simultaneous retrieval of NO and NO_2_ concentrations in UV-DOAS measurements. Its main novelty is the integration of a gas-specific dual-branch multi-scale convolution structure with a cross-attention mechanism to enhance the extraction and discrimination of overlapping absorption features of NO and NO_2_. The main contributions of this work are summarized as follows:

- A comprehensive UV-DOAS dataset and preprocessing pipeline are established. Spectral data are collected from single-component NO, single-component NO_2_, and mixed gases with various concentration combinations. The preprocessing pipeline consists of wavelength-range selection, differential optical absorption processing, Savitzky–Golay filtering, Z-score normalization, and tensor transformation, resulting in standardized training, validation, and test datasets.
- The MDIAN model is developed with a gas-specific dual-branch multi-scale convolution structure and a cross-attention-based feature interaction mechanism as the main architectural innovations. Bi-LSTM-based wavelength sequence modeling is further incorporated to aggregate contextual information along the spectral dimension. This integrated design helps mitigate spectral overlap interference and improve simultaneous concentration retrieval accuracy.
- The effectiveness of the proposed model is comprehensively validated through comparative experiments, ablation studies, stability analysis, and real-time performance evaluation. The results demonstrate that MDIAN achieves superior predictive accuracy compared with traditional methods and baseline deep learning models, while satisfying the requirements of real-time industrial monitoring.

The remainder of this paper is organized as follows. Section 2 introduces the experimental setup and dataset construction, including the principles of UV-DOAS, the spectral acquisition system, the data preprocessing pipeline, and dataset partitioning. Section 3 presents the overall architecture of the MDIAN model and the design of its components. Section 4 reports the experimental results and analysis, including model comparisons, ablation studies, stability analysis, and real-time performance evaluation. Finally, Section 5 concludes this work and outlines future research directions.

## 2. Experimental Setup and Dataset Construction

### 2.1. Beer–Lambert Law

**Figure 1:**
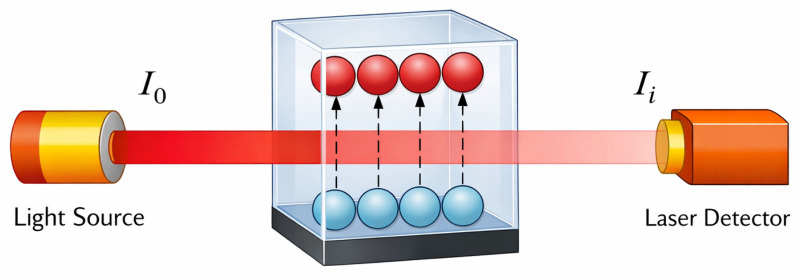
Schematic illustration of the Beer–Lambert law.

The Beer–Lambert law is a fundamental principle describing the attenuation of light intensity due to absorption by a medium [24,25], and it serves as the theoretical foundation of ultraviolet differential optical absorption spectroscopy (UV-DOAS) [26,27]. It describes the relationship between the incident and transmitted light as a beam passes through a homogeneous, non-scattering absorbing medium. The law includes two parts: Lambert’s law, which indicates that absorbance is proportional to the optical path length, and Beer’s law, which indicates that absorbance is proportional to the concentration of the absorbing species. The basic principle is shown in Figure 1.

When a collimated monochromatic light beam with wavelength λ propagates perpendicularly through a homogeneous gaseous medium, the attenuation of light intensity follows the Beer–Lambert law and can be expressed as:

(1) $$I(\lambda )=I_{0}(\lambda )exp[-\sigma (\lambda )cL]$$

where $I_{0}(\lambda )$ and I(λ) denote the incident and transmitted light intensities, respectively; σ(λ) is the absorption cross-section of the gas molecules (cm^2^ molecule^−1^); c is the number density of the gas (molecules cm^−3^), which can be converted to volume mixing ratio (ppm) under specified thermodynamic conditions; and L is the effective optical path length (cm).

In practical applications, the absorption characteristics are commonly described in terms of absorbance A and transmittance T, defined as:

(2) $$A=ln\left(\frac{I_{0}(\lambda )}{I(\lambda )}\right)=\sigma (\lambda )cL$$

(3) $$T=\frac{I(\lambda )}{I_{0}(\lambda )}=exp[-\sigma (\lambda )cL]$$

The absorbance A exhibits a linear relationship with the gas concentration c, which forms the basis for quantitative analysis using absorption spectroscopy [28]. By measuring the absorbance and combining Equations (1)–(3) with known absorption cross-sections and optical path length, the concentration of the target gas can be directly determined.

### 2.2. UV-DOAS Technique

However, in practical atmospheric environments and industrial emission monitoring, the ideal conditions assumed by the Beer–Lambert law are rarely fully satisfied. During light propagation, radiation is not only absorbed by target gases but also attenuated by Rayleigh scattering from atmospheric molecules and Mie scattering from aerosols. In addition, the absorption spectra of multiple gas species often overlap, making it difficult to directly distinguish the contribution of each component. Other factors, including light source fluctuations, temperature variations, and detector noise, may further introduce measurement uncertainties. Therefore, the direct application of the Beer–Lambert law to multi-component gas analysis remains limited.

To address these limitations, ultraviolet differential optical absorption spectroscopy (UV-DOAS) has been developed. Its core principle is to separate the total absorption into slowly varying and rapidly varying wavelength-dependent components. By removing the slowly varying component, the differential absorption signal containing characteristic molecular absorption features can be extracted, thereby suppressing scattering effects and background interference [29,30].

In UV-DOAS measurements, the optical density (OD), defined as the absorbance, is first introduced as:

(4) $$OD(\lambda )=ln\left(\frac{I_{0}(\lambda )}{I(\lambda )}\right)$$

The optical density includes contributions from both gas absorption and all scattering and attenuation processes [31]. Since Rayleigh and Mie scattering vary slowly with wavelength, and the broadband absorption of certain gases (e.g., oxygen and water vapor) also exhibits slow variation, the optical density can be decomposed into a slowly varying component (ODS) and a differential component (DOD):

(5) OD(λ)=ODS(λ)+DOD(λ)

The slowly varying component is typically obtained using low-order polynomial fitting:

(6) ODS(λ)=Polynomial(OD(λ))

In practice, third or fourth order polynomials are commonly used to approximate the contributions of scattering and broadband absorption. The DOD is then obtained by subtracting the slowly varying component from the total OD:

(7) DOD(λ)=OD(λ)−ODS(λ)

The DOD contains only the narrow-band absorption features of the target gases and exhibits a linear relationship with gas concentration:

(8) $$DOD(\lambda )=L\sum_{i}\sigma_{i}^{\prime }(\lambda )\;c_{i}$$

where $\sigma_{i}^{\prime }(\lambda )$ denotes the differential absorption cross-section of the i-th gas species, and $c_{i}$ is its concentration [32]. By fitting the measured DOD to the reference differential absorption cross-sections, the concentrations of multiple gas components can be simultaneously retrieved.

### 2.3. Spectral Acquisition

**Figure 2:**
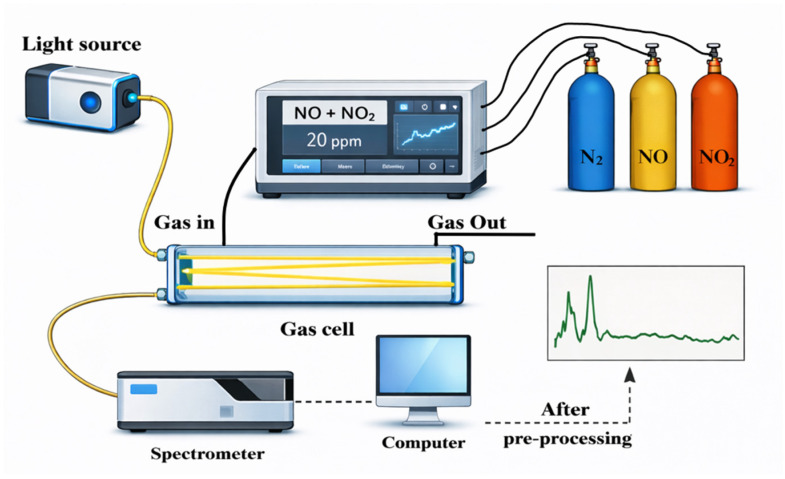
Flowchart of UV-DOAS gas detection.

The experimental system consists of a computer, an ultraviolet light source (xenon lamp), a dynamic gas dilution system, a spectrometer (M2-UV3, spectral resolution: 0.173 nm, Mooncell, Shenzhen, China), an absorption cell (optical path length: 12 m), standard gas cylinders, and an exhaust gas treatment unit [33,34]. In this study, high-purity N_2_ (99.999%), standard NO (50 ppm), and standard NO_2_ (50 ppm) were used. The target gas mixtures were generated by diluting these standard gases using a three-channel high-precision dynamic gas dilution system. The experimental setup is illustrated in Figure 2. The dynamic gas dilution system, equipped with mass flow controllers (accuracy: ±1% F.S.), was calibrated by a nationally certified institution to ensure high precision and traceability of gas flow control. All standard gases were stored in high-pressure cylinders, each accompanied by an individual certificate of analysis (CoA), which provides detailed information on gas composition and associated uncertainties.

Before each measurement, high-purity nitrogen (N_2_) was introduced at a flow rate of 500 mL min^−1^ for 5 min to purge the gas lines and eliminate residual gases. During formal measurements, the prepared gas mixtures were introduced into the absorption cell at a flow rate of 500 mL min^−1^ with a stabilization time of 3 min. The light emitted from the source was transmitted to the absorption cell via an optical fiber and formed an effective optical path length of 12 m through a White-type multiple-reflection configuration. The spectrometer then acquired the spectral signals, which were continuously recorded by the host computer. The wavelength range of 200–497 nm was selected for preprocessing and model input. This range, which is compatible with the xenon lamp and M2-UV3 spectrometer, captures the main UV absorption cross-sections of NO and NO_2_. A narrower range would omit useful features, while a broader range would introduce noise and increase computational cost. The spectra were processed directly using the original wavelength sampling points of the M2-UV3 spectrometer within the selected wavelength range of 200–497 nm. To ensure a stable measurement environment, the temperature inside the gas absorption cell was maintained at 50 °C using a proportional–integral–derivative (PID) controller, while the internal temperature of the spectrometer was stabilized at 45 °C. This configuration effectively minimizes the influence of ambient temperature fluctuations on absorption cross-sections and baseline drift. During the experiments, all gas lines were made of polytetrafluoroethylene (PTFE) with an inner diameter of 1/4 inch, and the gas system was maintained at atmospheric pressure.

To ensure the diversity and representativeness of the dataset, the concentration configurations covered the concentration range of 1 to 20 ppm [35]. First, single-component gases of NO and NO_2_ were prepared with concentrations ranging from 1 to 20 ppm. The concentrations were tested sequentially from low to high with a step size of 1 ppm. For each concentration level, 500 spectra were collected for NO and 500 spectra for NO_2_, resulting in a total of 20,000 single-gas spectral samples. Subsequently, 40 mixed-gas concentration combinations were prepared within the concentration range of 1–20 ppm for both NO and NO_2_. The mixed-gas dataset consisted of two subsets: an equal-concentration series, where the concentrations of NO and NO_2_ were both increased from 1 to 20 ppm, and an inverse-gradient series, where the NO concentration increased from 1 to 20 ppm while the NO_2_ concentration decreased from 20 to 1 ppm. This design covers both balanced mixtures and ratio-varying mixtures, enabling the evaluation of the model under different spectral-overlap and cross-interference conditions. The detailed concentration combinations are listed in Table 1. For each concentration combination, 500 spectra were acquired, yielding a total of 20,000 mixed-gas spectral samples. In addition, to obtain a stable reference light intensity $I_{0}(\lambda )$, 500 spectra of high-purity nitrogen (N_2_) were recorded under identical experimental conditions. The average of these spectra was taken as the reference spectrum, which was used for subsequent DOD calculation. The composition of the spectral dataset used in this study is summarized in Table 2, with a total of 40,500 raw spectral samples collected.

### 2.4. Data Preprocessing

**Figure 3:**
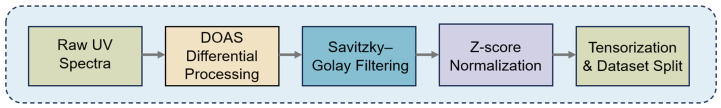
Data preprocessing pipeline.

To improve the consistency of the input spectra and enhance the stability of model training, the collected raw ultraviolet spectra were subjected to a unified preprocessing procedure [36], including differential optical absorption processing, Savitzky–Golay filtering, Z-score normalization, and tensor transformation, as illustrated in Figure 3. Through this pipeline, the raw intensity signals were transformed into standardized differential features suitable for deep learning-based modeling.

**Figure 4:**
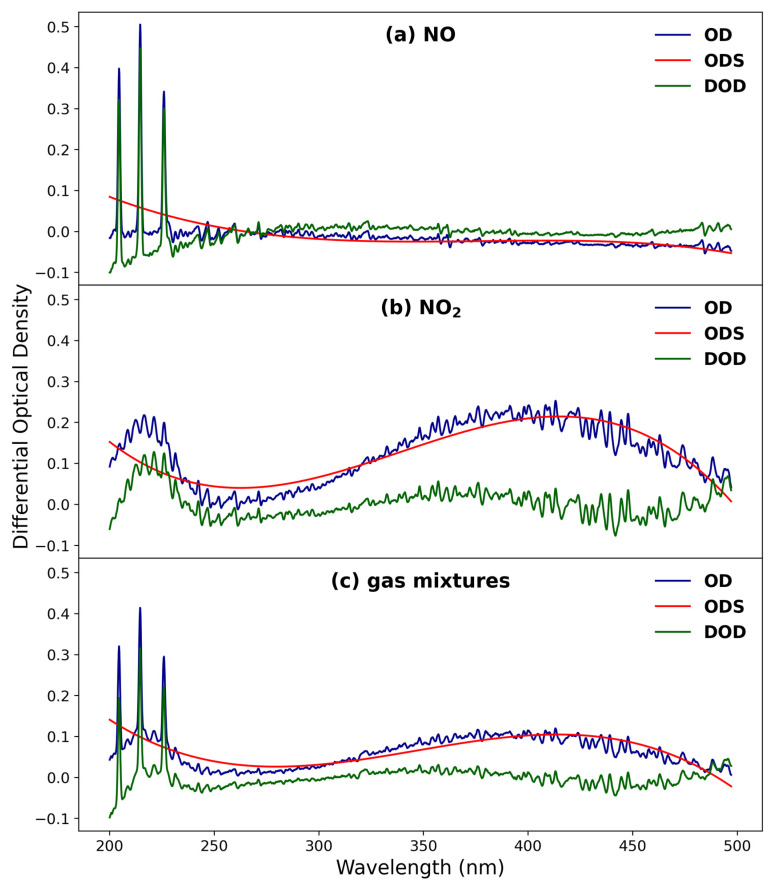
OD, ODS, and DOD spectra of different gases: (**a**) NO; (**b**) NO_2_; (**c**) gas mixtures.

First, the spectra were subjected to differential processing based on the DOAS principle. Specifically, according to Equations (4)–(8), the raw transmission spectra were first transformed into the optical density domain, after which the slowly varying background components with respect to wavelength were removed. In this study, a third-order polynomial was used to fit the slowly varying component of the OD spectrum for ODS extraction, because it provides sufficient flexibility to describe the smooth baseline variation while avoiding excessive fitting of narrow-band absorption features. This process yields differential absorption signals that retain only the narrow-band absorption features. In this study, the DOD spectra obtained after differential optical absorption processing were used as the basis for the final model input, rather than the raw intensity spectra or the original OD spectra. The DOAS differential processing results for NO, NO_2_, and their mixtures are presented in Figure 4. As shown, the differential processing effectively suppresses broadband scattering, baseline drift, and other slowly varying background contributions, thereby enhancing the fine spectral structures associated with molecular absorption of the target gases.

**Figure 5:**
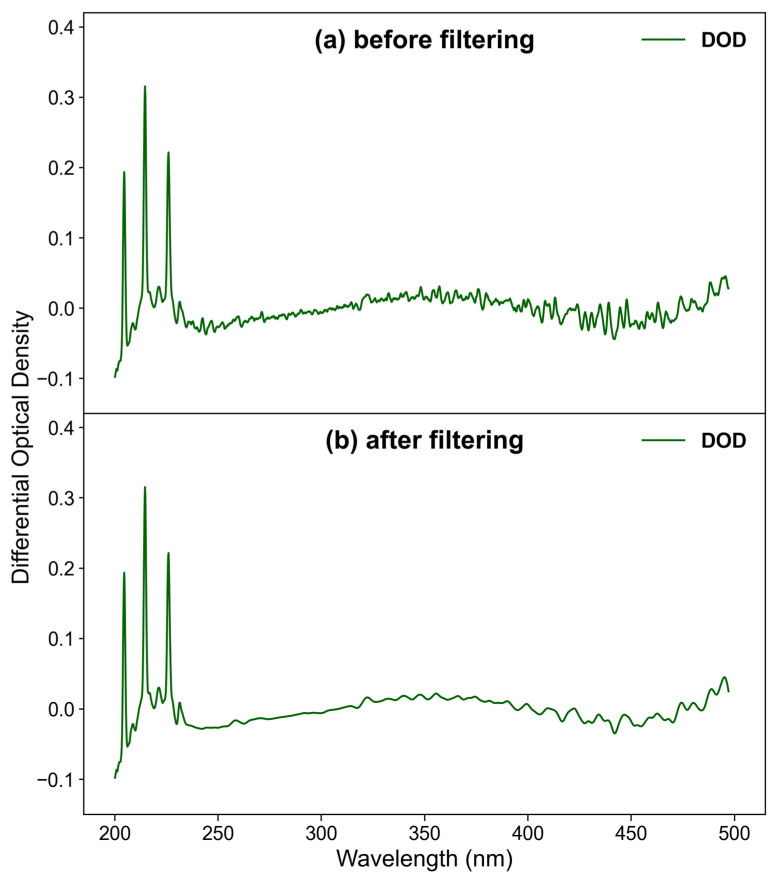
Comparison of spectra before and after Savitzky–Golay filtering.

After differential processing, Savitzky–Golay filtering was applied to smooth the spectra, aiming to suppress high-frequency random noise and improve the signal-to-noise ratio and stability of the input signals [37]. The filter parameters were set as a window length of ω=15 and a polynomial order of p=3. Considering the spectral resolution of 0.173 nm, the 15-point window corresponds to an approximately 2.6 nm smoothing interval. This setting provides a balance between noise suppression and feature preservation. A smaller window may retain excessive random noise, whereas a larger window may smooth out useful narrow-band absorption features. The third-order polynomial was used to preserve local spectral curvature while avoiding excessive fitting of random noise. A comparison of the spectra before and after filtering is shown in Figure 5, demonstrating that this parameter configuration effectively reduces noise while preserving the peak positions, bandwidths, and local variation trends of the spectral features.

On this basis, sample-wise Z-score normalization was applied to each DOD spectrum, where the mean and standard deviation were calculated from the wavelength points of the individual spectrum itself. This step did not use global statistics from the full dataset or information from the validation or test sets, thereby avoiding data leakage during normalization. It was used to mitigate the adverse effects of variations in overall signal amplitude across different samples on model training. This step ensures that all samples are brought to a comparable numerical scale, thereby improving the convergence stability during network training. Moreover, it encourages the model to focus on spectral shape variations and internal structural differences, rather than relying on absolute intensity levels.

After the above preprocessing procedures, the processed spectra were organized into input tensors with a unified format and paired with the corresponding NO and NO_2_ concentration labels. The comparative evaluation was conducted using the dataset partitioning strategy described in Section 2.5. In addition, to further examine the possible influence of repeated spectra measured under the same concentration condition, a concentration-condition-level grouped five-fold cross-validation strategy was introduced as an additional evaluation.

### 2.5. Dataset Partitioning

To train and evaluate the proposed MDIAN model, the collected spectral dataset was appropriately partitioned into training, validation, and test sets. A well-designed partitioning strategy is essential for ensuring the generalization capability of the model: the training set is used for parameter learning and optimization; the validation set is used for hyperparameter tuning, model selection, and implementation of the early-stopping strategy; and the test set, which consists of entirely unseen data, is used for the final objective evaluation of model performance.

**Figure 6:**
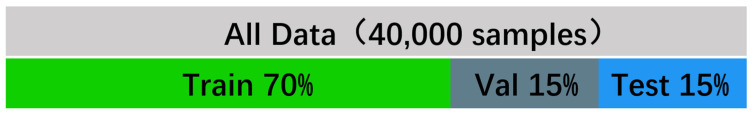
Dataset split configuration for training, validation and testing.

The dataset partitioning followed the principles below. First, random shuffling was applied to promote an approximately independent and identically distributed split of samples and to minimize potential systematic bias introduced by the acquisition order or batch effects. Second, a stratified sampling strategy was adopted to ensure that the training, validation, and test sets each contained samples of single-component NO, single-component NO_2_, and mixed gases, while preserving the concentration distribution of the original dataset. As shown in Figure 6, the training, validation, and test sets were divided at a ratio of 7:1.5:1.5. This partitioning scheme ensures sufficient training data while retaining adequate validation and test samples for reliable model selection and performance evaluation.

To further examine whether the model performance was affected by repeated spectra measured under the same concentration condition, an additional concentration-condition-level grouped five-fold cross-validation was performed. In this evaluation, each supervised concentration condition was treated as one independent group. The supervised dataset contained 80 concentration conditions, including 20 NO-only conditions, 20 NO_2_-only conditions, and 40 mixture conditions. The 500 repeated spectra measured under the same concentration condition were assigned to the same fold and were never split across the training and test sets.

The 80 concentration-condition groups were divided into five folds. Each fold contained 16 groups. To ensure that each fold was representative, the grouping was performed in a stratified and balanced manner. Specifically, each fold contained four NO-only conditions, four NO_2_-only conditions, four equal-concentration mixture conditions, and four inverse-gradient mixture conditions.

Within each category, the concentration levels were selected as uniformly as possible across the 1–20 ppm range. This design avoided concentrating low-, medium-, or high-concentration conditions in a single fold. In each cross-validation run, four folds were used for model training, and the remaining fold was used as the test set. This process was repeated five times. Therefore, each fold was used once as the test set. When a validation set was required for model selection or early stopping, it was selected only from the training-side concentration groups, and the held-out test fold was not used during training, validation, or hyperparameter selection.

Therefore, all spectra in the test subset came from concentration conditions that were not included in the corresponding training subset. This grouped evaluation was used as an additional validation. It assessed the generalization capability of the proposed model to unseen concentration conditions.

## 3. Model Architecture

### 3.1. Overall Architecture of MDIAN

**Figure 7:**
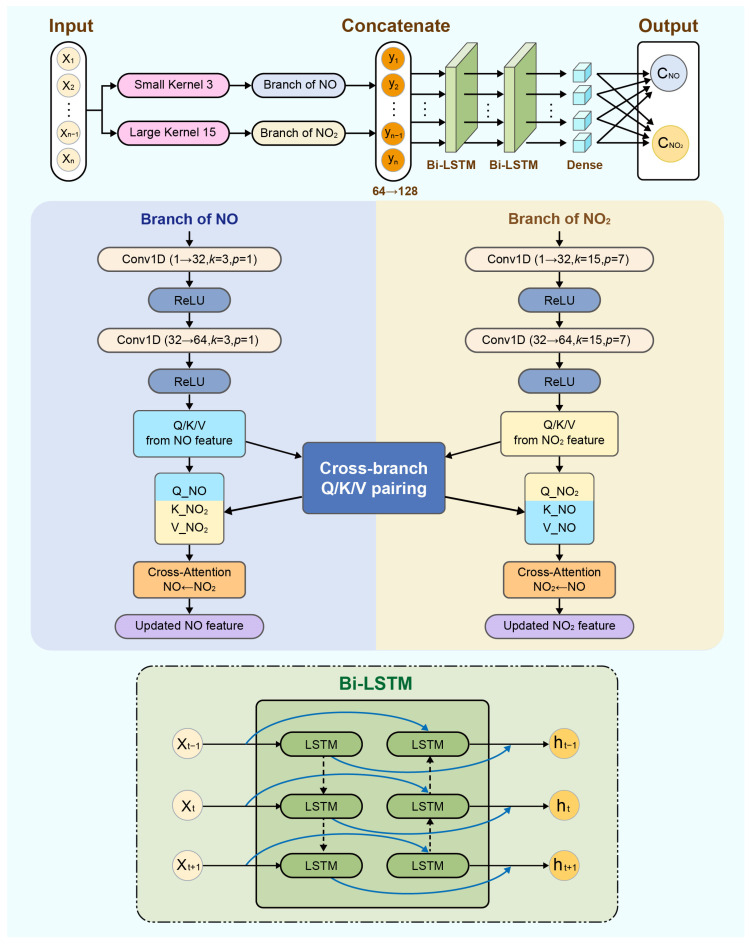
Schematic diagram of the MDIAN model architecture.

A multi-scale dual-branch interaction attention network (MDIAN) is proposed for the simultaneous retrieval of NO and NO_2_ concentrations in gas mixtures. The model is designed to address spectral overlap and nonlinear cross-interference in UV-DOAS measurements, which are difficult for traditional linear methods to handle. The overall architecture of the network is illustrated in Figure 7. The model takes the normalized DOD spectra, after Savitzky–Golay filtering and Z-score normalization, as input and outputs the concentrations of the two target gases. The overall processing pipeline consists of dual-branch convolutional feature extraction, cross-attention fusion, Bi-LSTM-based temporal aggregation, and fully connected regression output.

After tensor reshaping, the input spectra are processed by two convolutional branches with different kernel sizes, followed by bidirectional cross-attention, Bi-LSTM aggregation, and linear regression. The detailed design of these components is described in Section 3.2, Section 3.3 and Section 3.4.

Overall, this structure provides discriminative spectral representations for concentration retrieval in overlapping gas mixtures.

### 3.2. Dual-Branch Feature Extraction Module

The dual-branch feature extraction module is designed to capture scale-specific absorption features from the same input spectrum. Based on the DOD spectra, NO shows richer local narrow-band structures, whereas NO_2_ exhibits broader spectral profiles. Therefore, small kernels are used in the NO branch and larger kernels in the NO_2_ branch to learn complementary spectral features for subsequent fusion and concentration regression.

In implementation, the input spectra are first reshaped from $\left(\mathrm{batch},\mathrm{length},1\right)$ to $\left(\mathrm{batch},1,\mathrm{length}\right)$. Subsequently, the NO branch consists of two successive Conv1D layers followed by ReLU activations, with kernel sizes of 3 for both layers. The number of channels is increased from 1 to 32, and then from 32 to 64. Similarly, the NO_2_ branch is composed of two Conv1D layers with ReLU activations, but with kernel sizes of 15 for both layers, while following the same channel expansion strategy as the NO branch. Both branches employ symmetric padding to preserve the sequence length, resulting in feature representations of size $\left(\mathrm{batch},64,\mathrm{length}\right)$.

No pooling operation is used during convolutional feature extraction. Instead, sequence-length-preserving convolution is adopted for feature encoding. This design helps retain narrow-band absorption features and wavelength-position information for subsequent cross-attention and Bi-LSTM modeling.

### 3.3. Cross-Attention Fusion Module

To enhance the model’s ability to represent inter-species differences within overlapping absorption regions, a bidirectional cross-attention mechanism is introduced after the dual-branch convolutional encoding stage [38]. First, the feature tensors of the two branches are transposed from $\left(\mathrm{batch},64,\mathrm{length}\right)$ to $\left(\mathrm{batch},\mathrm{length},64\right)$, such that each wavelength position corresponds to a 64-dimensional feature vector. Subsequently, linear projections are applied to generate the Query, Key, and Value representations, followed by bidirectional feature interaction.

Specifically, when updating the NO branch, the NO features are used as the Query, while the NO_2_ features serve as the Key and Value. Conversely, when updating the NO_2_ branch, the same procedure is performed in the opposite direction. The cross-attention is computed using the scaled dot-product form:

(9) $$Z_{NO\leftarrow NO_{2}}=softmax\left(\frac{Q_{NO}K_{NO_{2}}^{T}}{\sqrt{d_{k}}}\right)V_{NO_{2}}$$

where Z denotes the attention-enhanced feature representation, Q, K, and V represent the Query, Key, and Value, respectively, and $d_{k}$ is the dimensionality of the key vectors. The attention output is then added to the original branch features through a residual connection. This allows complementary information from the other branch to be incorporated while preserving the original convolutional features.

Unlike simple concatenation or element-wise addition, cross-attention adaptively performs information exchange according to feature correlations. As a result, each branch can reference the response patterns of the other branch at adjacent or corresponding wavelength positions while modeling its own spectral features.

### 3.4. Temporal Feature Aggregation and Regression Output

After cross-attention updating, the features from the two branches are concatenated along the feature dimension, forming a fused representation of size $\left(\mathrm{batch},\mathrm{length},128\right)$. This representation contains both multi-scale convolutional features and cross-branch interaction information. A bidirectional long short-term memory (Bi-LSTM) network is then introduced for temporal aggregation while preserving the wavelength order.

In implementation, the input dimension of the Bi-LSTM is 128, with a hidden dimension of 128 and a network depth of two layers. A bidirectional structure is adopted, resulting in an output of size $\left(\mathrm{batch},\mathrm{length},256\right)$. Subsequently, the output at the final time step is taken as the sequence-level global representation and fed into a linear layer, Linear(256→2), to simultaneously predict the concentrations of NO and NO_2_.

The adoption of Bi-LSTM is motivated by the fact that UV-DOAS spectra are inherently one-dimensional sequences ordered by wavelength, in which strong continuity and contextual dependencies exist across different wavelength points. Compared with simple global averaging, Bi-LSTM more effectively preserves the sequential structure and captures long-range wavelength dependencies.

### 3.5. Evaluation Metrics

In this study, the mean squared error (MSE) was employed as the loss function for the regression task, while the mean absolute error (MAE), coefficient of determination ($R^{2}$), and root mean squared error (RMSE) were used as evaluation metrics [39,40]. Among them, MSE and RMSE are more sensitive to large deviations, whereas MAE reflects the average magnitude of absolute prediction errors. Smaller values of these metrics indicate closer agreement between the predicted and true values. In contrast, $R^{2}$ is used to evaluate the goodness of fit of the model, with values closer to 1 indicating better fitting performance. Their mathematical expressions are listed in Table 3.

## 4. Results and Analysis

### 4.1. Comparative Model Evaluation

To validate the effectiveness of the proposed MDIAN model for the simultaneous retrieval of NO and NO_2_ concentrations in gas mixtures, a standard DOAS least-squares method (LSM), support vector regression (SVR), convolutional neural networks (CNN), long short-term memory networks (LSTM), and a combined CNN + LSTM model were selected for comparison. LSM was introduced as the conventional spectroscopy baseline, while SVR was retained as a representative machine-learning baseline. All models were evaluated under the same dataset partitioning scheme and evaluation metric framework to ensure a fair comparison.

As shown in Table 4, the retrieval accuracy of NO and NO_2_ exhibits a consistent improvement trend as the methods evolve from the conventional DOAS least-squares method to machine-learning and deep-learning approaches. As the standard spectroscopy baseline, LSM provides a physically interpretable concentration retrieval method based on least-squares fitting. However, it produces the largest prediction errors and the lowest R^2^ values among all compared methods, indicating that the conventional linear fitting framework has limited capability in handling severe spectral overlap and cross-interference between NO and NO_2_. By introducing kernel mapping, SVR enhances nonlinear modeling capability and thus outperforms LSM overall. CNN is effective in extracting local spectral features, while LSTM captures contextual dependencies along the wavelength dimension. The combined CNN + LSTM model leverages the strengths of both, leading to further improvements in prediction accuracy. In contrast, the proposed MDIAN model integrates multi-scale dual-branch convolution, cross-attention fusion, and Bi-LSTM-based wavelength sequence modeling in a unified framework. This enables simultaneous extraction of narrow-band absorption details, broad spectral profile information, and inter-species coupling features. Compared with the best-performing deep learning comparison model (CNN + LSTM), MDIAN achieves additional reductions in MAE of 42.9% for NO and 38.6% for NO_2_, demonstrating superior discriminative capability and higher regression accuracy under complex overlapping spectral conditions.

**Figure 8:**
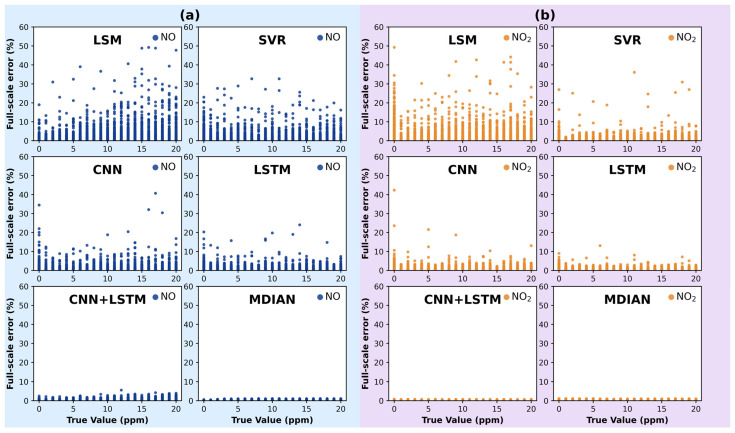
Full-scale prediction error distributions of different models: (**a**) NO; (**b**) NO_2_.

A more detailed view is provided by the full-scale error distributions in Figure 8. Although the errors of all comparative models are already maintained at relatively low levels for most samples, a small number of outliers with comparatively large errors can still be observed. By contrast, the error distribution of MDIAN is more concentrated and exhibits smaller overall fluctuations, indicating that it not only achieves higher average prediction accuracy but also provides better stability and robustness.

To further address the possible influence of repeated spectra under the same concentration condition, a concentration-condition-level grouped five-fold cross-validation was conducted. The results are shown in Table 5. In this evaluation, all 500 repeated spectra from the same concentration condition were kept in the same fold. Therefore, the spectra in each test fold came from concentration conditions that were not included in the corresponding training folds. The proposed MDIAN achieved stable performance across the five grouped folds. For NO retrieval, the MAE values ranged from 0.072 to 0.079 ppm, and the R^2^ values ranged from 0.9997 to 0.9998. For NO_2_ retrieval, the MAE values ranged from 0.061 to 0.067 ppm, and the R^2^ values ranged from 0.9997 to 0.9998.

These results are highly consistent with those obtained using the original dataset partitioning strategy. More importantly, the grouped evaluation avoided the situation in which repeated spectra from the same concentration condition appeared in both the training and test sets. Therefore, the high prediction accuracy of MDIAN was not mainly caused by near-duplicate spectra shared between the training and test sets. Instead, the results indicate that the proposed model learned concentration-related differential absorption features and showed good generalization capability to unseen concentration conditions within the investigated concentration range.

It should be noted that this grouped cross-validation also evaluated the model on concentration combinations that were not included in the corresponding training folds. In particular, each test fold contained unseen equal-concentration and inverse-gradient mixed-gas combinations. Therefore, the additional grouped evaluation further examined the ability of MDIAN to generalize to mixed-gas concentration pairs outside the training subset.

### 4.2. Ablation Study

**Figure 9:**
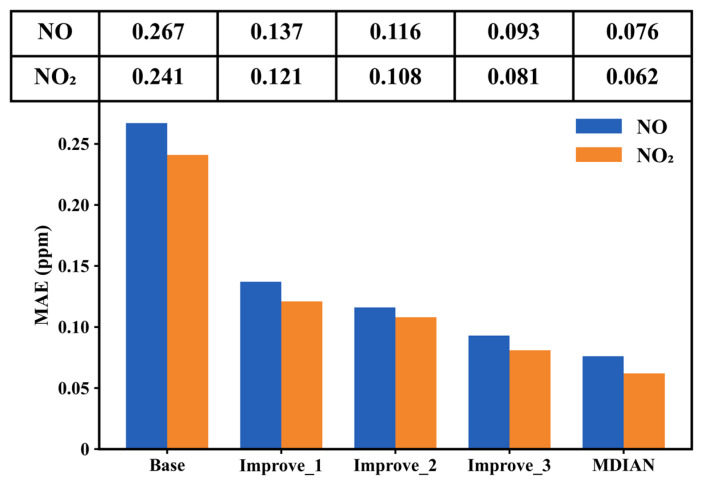
MAE comparison of different ablation models.

To systematically evaluate the contributions of each core component in MDIAN, we designed five groups of progressively cumulative ablation experiments. The configurations of these models are summarized in Table 6, and all variants were trained under the same training strategy and hyperparameter settings to ensure a fair comparison. To avoid ambiguity, the attention module in Improve_2 refers to self-attention applied within the single-branch spectral feature representation. It is used to enhance the weighting of informative wavelength regions under the single-branch setting. In contrast, the interaction attention module in Improve_3 refers to bidirectional cross-attention between the two branches. It enables feature interaction between the NO-related and NO_2_-related branches and is different from the self-attention used in Improve_2. The ablation results are presented in Figure 9. The Base model, which consists only of a single-branch convolutional module followed by a fully connected layer, exhibits the largest prediction errors, indicating that relying solely on single-branch convolution is insufficient to fully characterize the complex overlapping information in differential spectra of gas mixtures. After introducing Bi-LSTM, the MAE of Improve_1 shows a significant reduction compared with the Base model (with decreases of 48.7% for NO and 49.8% for NO_2_), demonstrating that contextual dependencies along the wavelength dimension play a critical role in concentration retrieval. With the further incorporation of an attention mechanism, Improve_2 achieves additional error reduction, suggesting that adaptive weighting of key spectral regions enhances the model’s ability to extract informative absorption features. Under the single-branch setting, to isolate and evaluate the contribution of the attention mechanism itself, a self-attention module is introduced as a replacement for comparative analysis.

When the model is extended from a single-branch to a dual-branch architecture with the incorporation of cross-attention, the performance of Improve_3 is further enhanced. This indicates that explicitly separating features from different branches and enabling cross-branch information exchange can more effectively mitigate spectral coupling interference between NO and NO_2_. Building upon Improve_3, the full MDIAN model further integrates multi-scale convolutional design, where small convolutional kernels are employed in the NO branch and larger kernels in the NO_2_ branch, enabling differentiated multi-scale feature extraction. Experimental results show that the complete MDIAN achieves the lowest MAE for both NO and NO_2_ (0.076 ppm for NO and 0.062 ppm for NO_2_). This demonstrates that small convolutional kernels are more effective in capturing local narrow-band absorption details, while larger kernels are better suited for modeling broad spectral profile characteristics. The complementary combination of the two further enhances the model’s capability to represent complex overlapping spectra.

The ablation study was designed in a progressively cumulative manner. This design allows the contribution of each main component to be evaluated step by step under a consistent experimental framework. Compared with isolated and non-cumulative variants, the progressive setting provides a clearer view of how the model performance changes when key modules are sequentially introduced. It also avoids excessive structural changes between adjacent variants, making the performance differences easier to interpret. The consistent performance improvement observed across the ablation variants demonstrates the effectiveness and necessity of the main modules in the proposed architecture.

### 4.3. Stability Analysis

In addition to prediction accuracy, the practical applicability of the model in real detection scenarios also depends on the stability and sensitivity of its outputs. To this end, the uncertainty A and detection limit DL were adopted to further evaluate the MDIAN model. Their definitions are given in Equations (10) and (11):

(10) $$A=\sqrt{\frac{\sum_{i=1}^{n}(S_{i}-S^{\prime }{)}^{2}}{n(n-1)}}$$

(11) $$DL=K_{i}\delta_{0}C/S^{\prime }$$

where $S_{i}$ denotes the concentration value predicted in the i-th repeated measurement (ppm), $S^{\prime }$ denotes the average value of the repeated measurement results (ppm), and n is the number of repeated measurements. In this study, n=50. $K_{i}$ is the dimensionless confidence factor and was set to 3. This expression is equivalent to the standard 3σ/sensitivity convention, where the sensitivity is estimated as $S^{\prime }/C$. C is the standard concentration of the target gas (ppm), which was 10 ppm for both NO and NO_2_ in the repeated measurement experiment. $\delta_{0}$ denotes the standard deviation of the repeated measurement results (ppm) and was calculated from the 50 consecutive predicted concentrations for each gas. Specifically, for each target gas, the 50 predicted concentrations were first used to calculate $S^{\prime }$, and the corresponding standard deviation was then taken as $\delta_{0}$. The uncertainty and detection limit were calculated separately for NO and NO_2_, and the detection limit is expressed in ppm.

**Figure 10:**
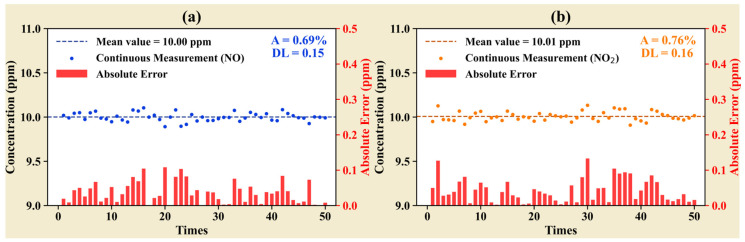
Statistical results of stability analysis: (**a**) NO; (**b**) NO_2_.

A mixed gas containing 10 ppm NO and 10 ppm NO_2_ was measured in 50 consecutive trials, and the results are presented in Figure 10. For NO detection, the uncertainty A and detection limit DL were 0.69% and 0.15 ppm; for NO_2_, the corresponding values were 0.76% and 0.16 ppm. It should be noted that these detection limits were statistically estimated from repeated measurements at 10 ppm and represent the estimated sensitivity of the system under the current experimental conditions. In the present study, the experimental validation was conducted within the concentration range of 1–20 ppm, while sub-ppm measurements were beyond the scope of the current experimental design. The uncertainty A for both gases is below 1%, indicating that the model output exhibits minimal fluctuations and thus demonstrates good repeatability and stability. In terms of the detection limit DL, both NO and NO_2_ remain at relatively low levels, with a difference of only 0.01 ppm. These results suggest that MDIAN exhibits good repeatability and estimated sensitivity for the simultaneous retrieval of NO and NO_2_ under the current experimental conditions.

To further evaluate the repeatability and estimated sensitivity of MDIAN across different concentration levels, four equal-concentration NO/NO_2_ mixtures were tested. The selected concentration levels were 1, 5, 10, and 20 ppm for both NO and NO_2_, representing near-zero, low, medium and high concentration levels within the investigated 1–20 ppm range. For each concentration level, 50 consecutive repeated measurements were performed.

The results are summarized in Table 7. For the 1 ppm NO/NO_2_ mixture, the uncertainty and detection limit were slightly higher than those obtained at higher concentrations. Specifically, the uncertainty values were 0.87% for NO and 0.95% for NO_2_, while the detection limits were 0.24 ppm and 0.27 ppm, respectively. This is reasonable because the absorption signal is weaker at the near-zero/low-concentration level, making the relative influence of prediction fluctuation more pronounced. As the concentration increased to 5, 10, and 20 ppm, both uncertainty and detection-limit values became more stable. For these three concentration levels, the uncertainty values remained below 0.80%, and the detection limits were approximately 0.15–0.16 ppm for both gases.

These results indicate that MDIAN maintains good repeatability across the investigated 1–20 ppm range. The slightly larger uncertainty and detection limit at 1 ppm reflect the expected behavior of low-concentration spectral retrieval, where weaker absorption signals lead to relatively larger fluctuations. Overall, the model shows stable uncertainty and detection-limit performance under low, medium, and high concentration conditions.

### 4.4. Real-Time Performance Analysis

**Figure 11:**
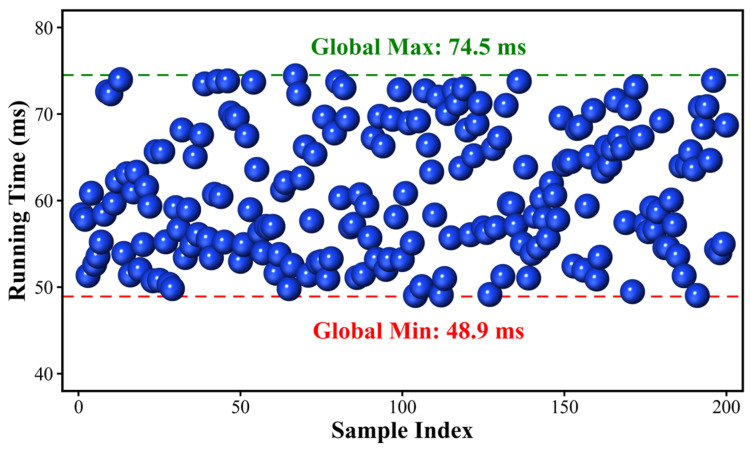
Real-time performance evaluation results.

To evaluate the real-time performance of the proposed MDIAN model in practical application scenarios, its inference speed was systematically assessed. In this study, the model was implemented in Python 3.13 and executed on a system equipped with an AMD Ryzen 9 8945H processor and an NVIDIA RTX 4060 GPU with CUDA acceleration. Figure 11 presents the model-side inference time statistics for 200 spectral samples randomly selected from the test set. Each sample was processed independently with a batch size of 1, and the forward-pass time from the preprocessed spectral tensor input to the NO/NO_2_ concentration output was recorded. The results show that the maximum inference time is 74.5 ms, while the minimum is 48.9 ms. These results indicate that the proposed method demonstrates strong real-time performance and can satisfy the requirements of real-time monitoring in practical industrial scenarios.

## 5. Conclusions

To address the spectral overlap and nonlinear cross-interference between NO and NO_2_ in UV-DOAS measurements, this study proposed MDIAN for simultaneous concentration retrieval in gas mixtures. A UV-DOAS experimental system and preprocessing pipeline were established to obtain normalized DOD spectra from single-component and mixed-gas samples. The proposed model combines gas-specific dual-branch convolution, cross-attention interaction, and Bi-LSTM-based wavelength-sequence aggregation to improve the representation of overlapping absorption features.

Experimental results show that MDIAN achieves the best performance among all compared models. The MAE values for NO and NO_2_ are 0.076 ppm and 0.062 ppm, respectively, and the corresponding R^2^ values reach 0.9998 for both gases. Ablation experiments confirm the contribution of the key modules, while stability and real-time tests demonstrate good repeatability and inference efficiency under the current experimental conditions.

In summary, the proposed MDIAN model effectively addresses the challenges of spectral overlap and nonlinear cross-interference in UV-DOAS-based concentration retrieval of gas mixtures, and demonstrates strong performance in terms of accuracy, stability, sensitivity, and real-time capability. Nevertheless, the present study was conducted using a controlled laboratory dataset, and field validation under more variable environmental and industrial conditions has not yet been performed. Although random shuffling, stratified sampling and cross-validation were adopted for model evaluation, independent external datasets are still needed to further assess the model’s generalization capability. Future work may focus on incorporating multi-component gas datasets under more complex operating conditions. Other gases with characteristic ultraviolet absorption features, such as SO_2_ and NH_3_, may also be included. This would help further investigate the model’s generalization ability and its performance in practical field applications.

## Figures and Tables

**Table 1:** Forty NO and NO_2_ concentration combinations used in the mixed-gas experiments.

Equal-Concentration Series						Inverse-Gradient Series					
NO.	NO	NO_2_	NO.	NO	NO_2_	NO.	NO	NO_2_	NO.	NO	NO_2_
1	1	1	11	11	11	21	1	20	31	11	10
2	2	2	12	12	12	22	2	19	32	12	9
3	3	3	13	13	13	23	3	18	33	13	8
4	4	4	14	14	14	24	4	17	34	14	7
5	5	5	15	15	15	25	5	16	35	15	6
6	6	6	16	16	16	26	6	15	36	16	5
7	7	7	17	17	17	27	7	14	37	17	4
8	8	8	18	18	18	28	8	13	38	18	3
9	9	9	19	19	19	29	9	12	39	19	2
10	10	10	20	20	20	30	10	11	40	20	1

**Note:** Concentrations are given in ppm. Combinations 1–20 represent the equal-concentration series, while combinations 21–40 represent the inverse-gradient series.

**Table 2:** Composition of the spectral dataset.

Gas Species	Concentration Range (ppm)	Number of Concentration Levels	Number of Replicates	Total Spectra
N_2_	—	1	500	500
NO	1–20	20	500	10,000
NO_2_	1–20	20	500	10,000
NO + NO_2_	1–20	40	500	20,000

**Table 3:** Loss function and evaluation metrics.

Category	Equation
Loss function	$MSE=\frac{1}{n}\sum_{i=1}^{n}{\left(y_{i}-{\hat{y}}_{i}\right)}^{2}$
Evaluation Criteria	$MAE=\frac{1}{n}\sum_{i=1}^{n}\left|y_{i}-{\hat{y}}_{i}\right|$
	$R^{2}=1-\frac{\sum_{i=1}^{n}{\left(y_{i}-{\hat{y}}_{i}\right)}^{2}}{\sum_{i=1}^{n}{\left(y_{i}-\bar{y}\right)}^{2}}$
	$RMSE=\sqrt{\frac{1}{n}\sum_{i=1}^{n}{\left(y_{i}-{\hat{y}}_{i}\right)}^{2}}$

**Note:** n denotes the number of samples, ${\hat{y}}_{i}$ denotes the predicted value of the i-th sample, $y_{i}$ denotes the true value of the i-th sample, and $\bar{y}$ denotes the mean of all true values.

**Table 4:** Comparative evaluation results of different models.

Model	Gas					
	NO			NO_2_		
	MAE	RMSE	$R^{2}$	MAE	RMSE	$R^{2}$
LSM	0.592	1.422	0.9538	0.582	1.211	0.9684
SVR	0.306	0.769	0.9865	0.184	0.539	0.9937
CNN	0.197	0.647	0.9904	0.160	0.314	0.9979
LSTM	0.158	0.320	0.9977	0.124	0.188	0.9992
CNN + LSTM	0.133	0.163	0.9994	0.101	0.102	0.9997
MDIAN	0.076	0.099	0.9998	0.062	0.084	0.9998

**Table 5:** Five-fold cross-validation results of MDIAN.

Fold	Training Subset Number	Test Subset Number	Gas			
			NO		NO_2_	
			MAE	$R^{2}$	MAE	$R^{2}$
1	2, 3, 4, 5	1	0.074	0.9998	0.061	0.9998
2	1, 3, 4, 5	2	0.079	0.9997	0.067	0.9998
3	1, 2, 4, 5	3	0.072	0.9998	0.064	0.9997
4	1, 2, 3, 5	4	0.075	0.9997	0.062	0.9998
5	1, 2, 3, 4	5	0.076	0.9997	0.063	0.9998

**Table 6:** Definition of ablation model groups and configurations.

Model	Multi-Scale	Dual-Branch	Interaction Attention	Bi-LSTM
Base	×	×	×	×
Improve_1	×	×	×	√
Improve_2	×	×	√	√
Improve_3	×	√	√	√
MDIAN	√	√	√	√

**Note:** √ denotes that this component is included in the model; while × denotes that this component is not included in the model.

**Table 7:** Uncertainty and detection limit results at different NO/NO_2_ concentration levels.

Concentration Level (ppm)	NO		NO_2_	
	A	DL (ppm)	A	DL (ppm)
1	0.87%	0.24	0.95%	0.27
5	0.72%	0.16	0.78%	0.16
10	0.69%	0.15	0.76%	0.16
20	0.69%	0.16	0.75%	0.15

## Data Availability

The data supporting this study’s findings are available from the corresponding author upon reasonable request.
